# Gut microbiota from voluntary exercised mice protects the intestinal barrier by inhibiting neutrophil extracellular trap formation

**DOI:** 10.1016/j.isci.2025.112763

**Published:** 2025-05-27

**Authors:** Beibei Zhu, Hao Wu, Han Zhang, Qi Song, Yong Xiao, Baoping Yu

**Affiliations:** 1 Department of Gastroenterology, Renmin Hospital of Wuhan University, Wuhan, Hubei 430060, China; 2 Key Laboratory of Hubei Province for Digestive System Diseases, Wuhan, Hubei 430060, China; 3 Department of Radiology, Taihe Hospital, Hubei University of Medicine, Shiyan, Hubei 442000, China

**Keywords:** Immunology, Microbiome

## Abstract

Ulcerative colitis is an inflammatory bowel disease characterized by impaired intestinal barrier function, dysregulated immune responses, and alterations in the gut microbiota. Excessive formation of neutrophil extracellular traps (NETs), driven by peptidyl arginine deiminase 4 (PAD4) activity, contributes to inflammation modulated by the gut microbiota. In this study, we used a mouse model of dextran sulfate sodium-induced colitis to investigate the effects of voluntary exercise and its underlying mechanisms. Exercise preconditioning attenuated colitis severity, maintained intestinal barrier integrity, normalized gut microbiota composition, and suppressed NET formation. PAD4 inhibition further enhanced these effects. By contrast, the depletion of the gut microbiota by antibiotics largely abolished the benefits of exercise. Additionally, fecal microbiota transplantation from exercised mice recapitulated these protective effects. These findings elucidate the interplay among exercise, gut microbiota, and PAD4-mediated NET formation. Targeting these pathways may offer promising therapeutic strategies for colitis.

## Highlights

• Voluntary exercise inhibited NET formation in acute colitis
• Gut microbiota depletion partially abolished the benefits of exercise
• Exercise-derived fecal microbiota transplantation replicated the protective effects

## Graphical abstract

**Figure:**
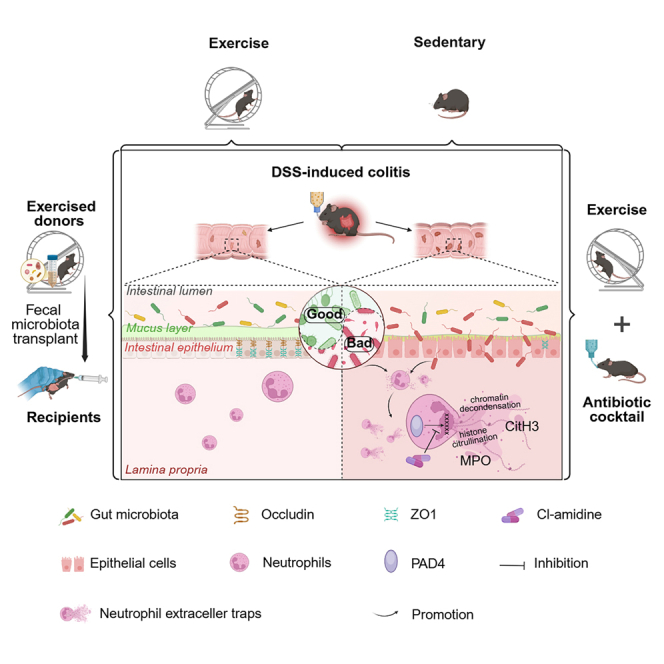

## Introduction

Ulcerative colitis (UC) is a form of inflammatory bowel disease (IBD) with an increasing global incidence and prevalence.^1^ Its pathogenesis remains unclear. UC arises from an abnormal immune response to the gut microbiota, triggered by the disruption of the intestinal mucosal barrier in genetically predisposed individuals.^2^^,^^3^ Current treatments focus on symptom management. However, UC is difficult to cure, prone to relapse, and carries the risk of malignancy, leading to low treatment efficacy, high recurrence rates, and significant healthcare costs.^4^^,^^5^ Given these challenges, effective interventions with minimal side effects need to be determined to prevent and manage UC. Exercise is considered one of the most important lifestyle strategies for patients with UC. Epidemiological studies have demonstrated that regular physical activity is associated with a reduced risk of UC.^6^^,^^7^^,^^8^ Moreover, exercise decreases the risk of active UC, alleviates symptoms, mitigates adverse drug reactions (such as those caused by 5-aminosalicylic acid), and lowers the risk of colorectal cancer.^9^^,^^10^^,^^11^ Additionally, moderate-intensity exercise reduces colonic inflammation by supporting intestinal barrier function.^12^

Gut microbiota dysbiosis is strongly associated with UC severity in clinical patients.^13^ It is well established that probiotic supplementation can reduce colitis and strengthen the intestinal barrier.^14^ Recent studies have suggested that exercise not only maintains the integrity of the intestinal barrier but also promotes gut microbiota diversity and health.^15^^,^^16^ However, most of these studies were observational and based on microbial sequencing, leaving the causal relationship among exercise, gut microbiota, and the intestinal barrier unclear. Thus, the present study aimed to address this gap by examining the effects of exercise on colonic inflammation and intestinal barrier function after gut microbiota depletion in post-exercise mice using antibiotics.

Although exercise is a promising intervention for UC, many patients show significantly reduced physical activity after diagnosis because of fatigue and weakness, limiting their ability to stay active.^17^^,^^18^ Therefore, identifying alternatives that offer similar benefits is a priority. In recent years, fecal microbiota transplantation (FMT) from healthy donors has gained attention as a way to restore gut microbiota in patients with IBD.^19^ Systematic reviews and meta-analyses of nonrandomized studies have shown that FMT can induce remission in UC and improve clinical and endoscopic outcomes.^20^^,^^21^ Some studies used FMT from exercise donors to replicate the effects of exercise on insulin resistance and hypertension in mice.^22^^,^^23^ However, studies on the effects of exercise-derived FMT on colitis remain limited. Our study evaluated the effects of exercise-derived FMT on colitis and compared its effects with those of a direct exercise intervention.

Additionally, the potential mechanisms of exercise are worth investigating. Neutrophils are the first immunocytes to respond to inflammation and are recruited to the inflamed intestinal mucosa to eliminate invading pathogens via phagocytosis, degranulation, and the release of neutrophil extracellular traps (NETs).^24^^,^^25^ NETs are fibrous web-like structures extruded by activated neutrophils and primarily composed of deoxyribonucleic acid, histones, myeloperoxidase (MPO), and neutrophil elastase.^25^ Although NET formation serves as an effective defense mechanism against pathogens, excessive release can damage host tissues.^26^ Recent studies have highlighted the pathological role of NETs in patients with UC, contributing to intestinal injury and prothrombotic tendencies.^27^^,^^28^ Additionally, NET-related proteins are significantly elevated in experimental colitis mouse models, and targeting NET formation or degradation restores the intestinal barrier function and improves colonic inflammation in these mice.^29^^,^^30^^,^^31^^,^^32^^,^^33^ Peptidyl arginine deiminase 4 (PAD4) is a critical enzyme in NET formation that catalyzes histone citrullination to induce chromatin decondensation.^34^ PAD4 inhibition reduces NET formation and ameliorates colonic inflammation in experimental models.^33^ Although the effects of exercise on NETs have been observed in various animal models of the disease, no study has specifically examined its role in colitis-associated NETs. Therefore, this study explored whether voluntary exercise could reduce NETs by regulating the gut microbiota to maintain the integrity of the intestinal mucosal barrier.

Our findings revealed that voluntary exercise preconditioning alleviated the severity of dextran sulfate sodium (DSS)-induced colitis, preserved intestinal barrier integrity, prevented microbiota dysbiosis, and diminished NET formation. However, these protective effects were largely abolished by antibiotic-induced gut microbiota depletion, underscoring the essential role of the microbiota in mediating these benefits. To confirm microbiota dependency, FMT from exercised donors replicated the protective effects, reinforcing the role of gut microbiota in exercise-induced colitis protection and NET regulation. Furthermore, the pharmacological inhibition of PAD4 enhanced the anti-inflammatory effects observed in the exercise group. Collectively, these results suggest that alterations in exercise-derived microbiota influence immune regulation and NET suppression, with PAD4 inhibition providing an additional therapeutic synergy. This study highlights the pivotal role of the gut microbiota in exercise-induced protection against colitis and proposes that exercise-derived FMT combined with targeted NET modulation may be a promising therapeutic strategy for UC.

## Results

To ensure the consistency and reproducibility of the voluntary exercise effects, we monitored the running activity of the exercised mice throughout the study. Table 1 presents the average daily running distance and total running time of the voluntary exercise group, confirming that the mice engaged in sustained physical activity. These data further support the idea that exercise-induced physiological changes contribute to the protection against colitis.

**Table 1:** Weekly observations of mice during exercise

Group	Variable	Feed weeks						
		W0	W1	W2	W3	W4	W5	W6
Voluntary exercise (*n* = 16)	weight (g)	19.31 ± 0.66	19.93 ± 0.86	20.98 ± 0.92	21.98 ± 0.96	22.86 ± 0.91	23.31 ± 0.91	23.95 ± 0.95
	running (10^4^ circles)	0	6.404 ± 0.75	7.567 ± 0.965	8.1 ± 0.76	7.53 ± 0.59	7.32 ± 0.83	7.82 ± 1.02
	food intake (g)	0	35.77 ± 2.6	35.40 ± 1.95	37.23 ± 2.13	36.52 ± 3.2	38.6 ± 3.32	38.83 ± 2.00
	water intake (mL)	0	36.85 ± 1.13	37.34 ± 1.64	38.75 ± 4.1	39.61 ± 4.51	40.59 ± 3.59	38.41 ± 3.29
Sedentary (*n* = 16)	weight (g)	19.23 ± 0.81	20.56 ± 0.98	21.73 ± 1.18	22.75 ± 1.37	23.93 ± 1.51	25.07 ± 1.67	25.42 ± 1.55
	food intake (g)	0	32.58 ± 0.64	34.09 ± 2.03	35.01 ± 1.69	35.29 ± 0.67	36.52 ± 0.84	35.59 ± 3.08
	water intake (mL)	0	36.09 ± 2.58	34.46 ± 1.71	37.16 ± 1.14	36.69 ± 1.13	37.39 ± 2.09	37.95 ± 1.36

### Intact gut microbiota was required for exercise-mediated protection against colitis

**Figure 1:**
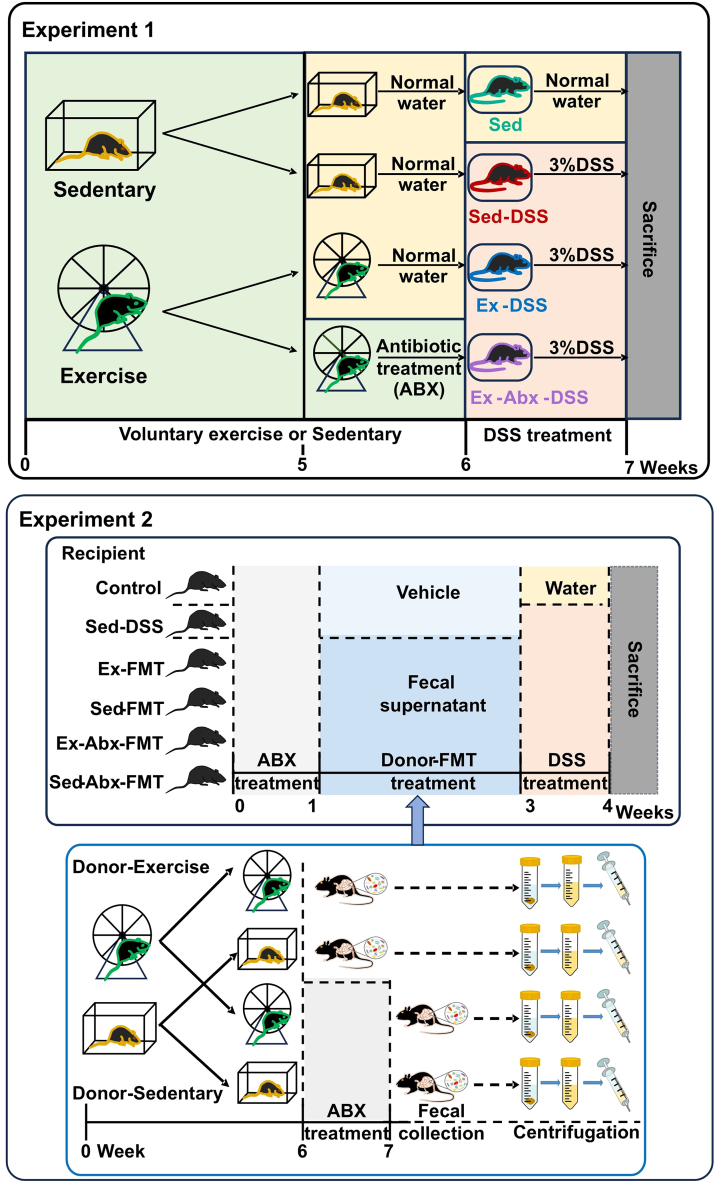
Overview of grouping and experimental procedures Experiment 1: outline of the voluntary exercise model, antibiotics-treated microbiota depletion model, and DSS-induced acute colitis model. Experiment 2: preparation of donor fecal suspensions and the process of fecal microbiota transplantation. DSS, dextran sulfate sodium; Sed, sedentary control; Sed-DSS, sedentary DSS-treated mice; Ex-DSS, exercised DSS-treated mice; Abx, antibiotics; FMT, fecal microbiota transplantation.

**Figure 2:**
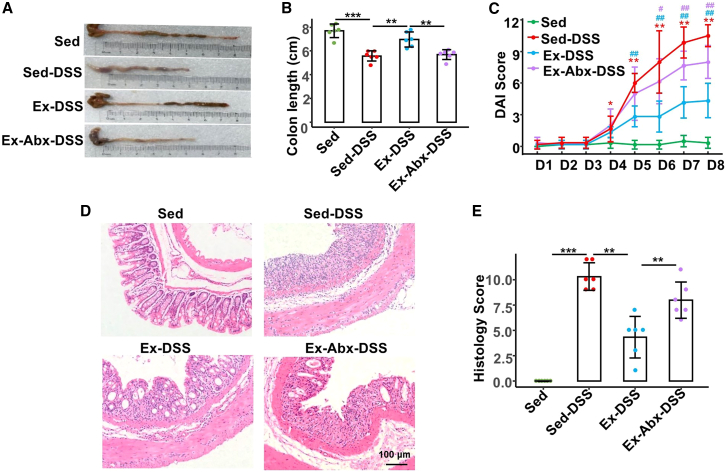
Exercise protects against DSS-induced colitis via a microbiota-dependent mechanism (A and B) Images and bar plot of colon length in mice. (C) Clinical DAI scores of each group. (D) Representative hematoxylin and eosin staining images of colonic sections at 200× magnification, scale bars: 100 μm. (E) Histological scores. Data are presented as means ± standard deviations, *n* = 6 per group, red ∗∗ and ∗ represent crosswise comparison between the Sed-DSS and Sed groups at *p* < 0.01 and *p* < 0.05, respectively; blue ^##^ and ^#^ represent crosswise comparison of the Ex-DSS and Sed-DSS groups with *p* < 0.01 and *p* < 0.05, respectively; purple ## and # represent crosswise comparison of the Ex-Abx-DSS and Ex-DSS groups with *p* < 0.01 and *p* < 0.05, respectively. DAI, disease activity index; DSS, dextran sulfate sodium; Sed, sedentary control; Sed-DSS, sedentary DSS-treated mice; Ex-DSS, exercised DSS-treated mice; Abx, antibiotics.

To determine the effects of voluntary exercise on the severity of colitis, we compared four groups as follows: sedentary control (Sed), sedentary DSS-treated mice (Sed-DSS), exercised DSS-treated mice (Ex-DSS), and exercised DSS-treated mice with antibiotic-induced microbiota depletion (Ex-Abx-DSS). For the experimental workflow, please see Experiment 1 in Figure 1 (located in the STAR Methods section). As shown in Figure 2, DSS treatment in sedentary mice caused marked colonic shortening (*p* < 0.001) (Figures 2A and 1B), elevated disease activity index (DAI) scores (*p* = 0.004) (Figure 2C), and severe histological damage (*p* < 0.001) (Figures 2D and 1E). By contrast, the exercised mice (Ex-DSS) exhibited significantly longer colons (*p* = 0.002), lower DAI scores (*p* = 0.005), and reduced tissue injury (*p* = 0.004). Importantly, when the gut microbiota was depleted (Ex-Abx-DSS), the protective effects of exercise were largely abolished (*p* = 0.003), DAI scores increased (*p* = 0.006), and pathological damage worsened (*p* = 0.002), similar to those in the Sed-DSS group. An intact microbial community may be essential for the exercise-induced mitigation of DSS-induced colonic inflammation.

### Voluntary exercise maintained intestinal barrier integrity in a microbiota-dependent manner

**Figure 3:**
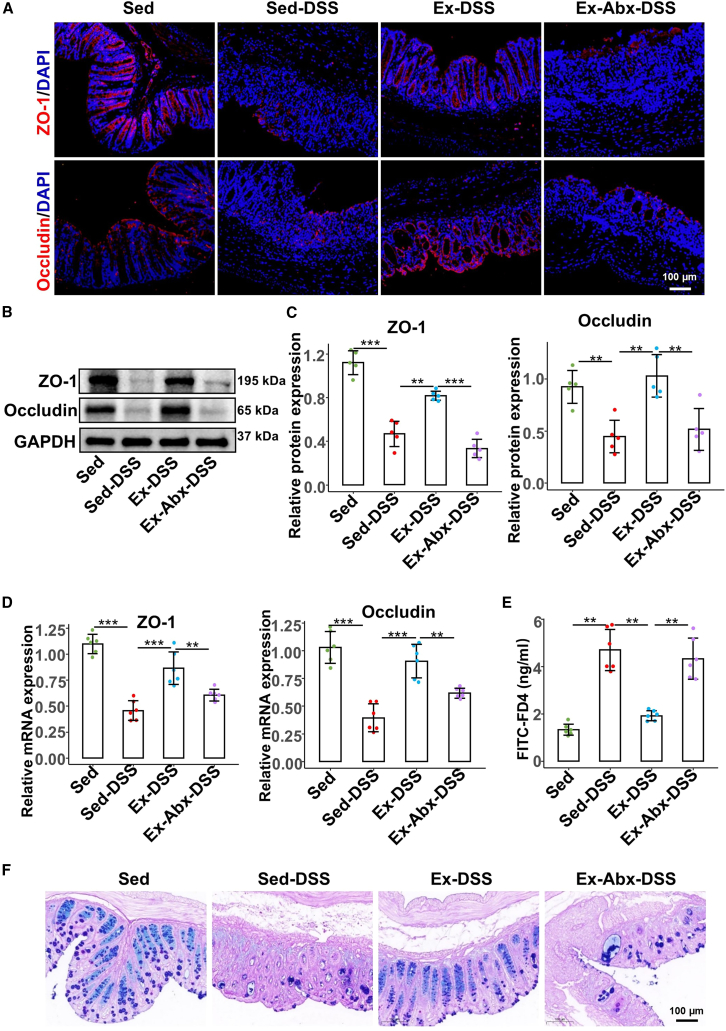
Voluntary exercise maintains the intestinal barrier function but is compromised by antibiotic-induced microbiota depletion (A) The distribution of ZO-1 and occludin in colonic tissues is observed by immunofluorescence staining, scale bars: 100 μm. (B and C) The protein levels of colonic ZO-1 and occludin were evaluated by western blotting (*n* = 5 per group). (D) The mRNA expression of colonic ZO-1 and occludin was examined by reverse-transcription polymerase chain reaction. (E) The intestinal permeability is analyzed via oral gavage of FITC-FD4. (F) The number of goblet cells in the colon is visualized by AB-PAS staining. Data are presented as means ± standard deviations, ∗∗*p* < 0.01, and ∗∗∗*p* < 0.001. DSS, dextran sulfate sodium; Sed, sedentary control; Sed-DSS, sedentary DSS-treated mice; Ex-DSS, exercised DSS-treated mice; Abx, antibiotics.

**Figure 4:**
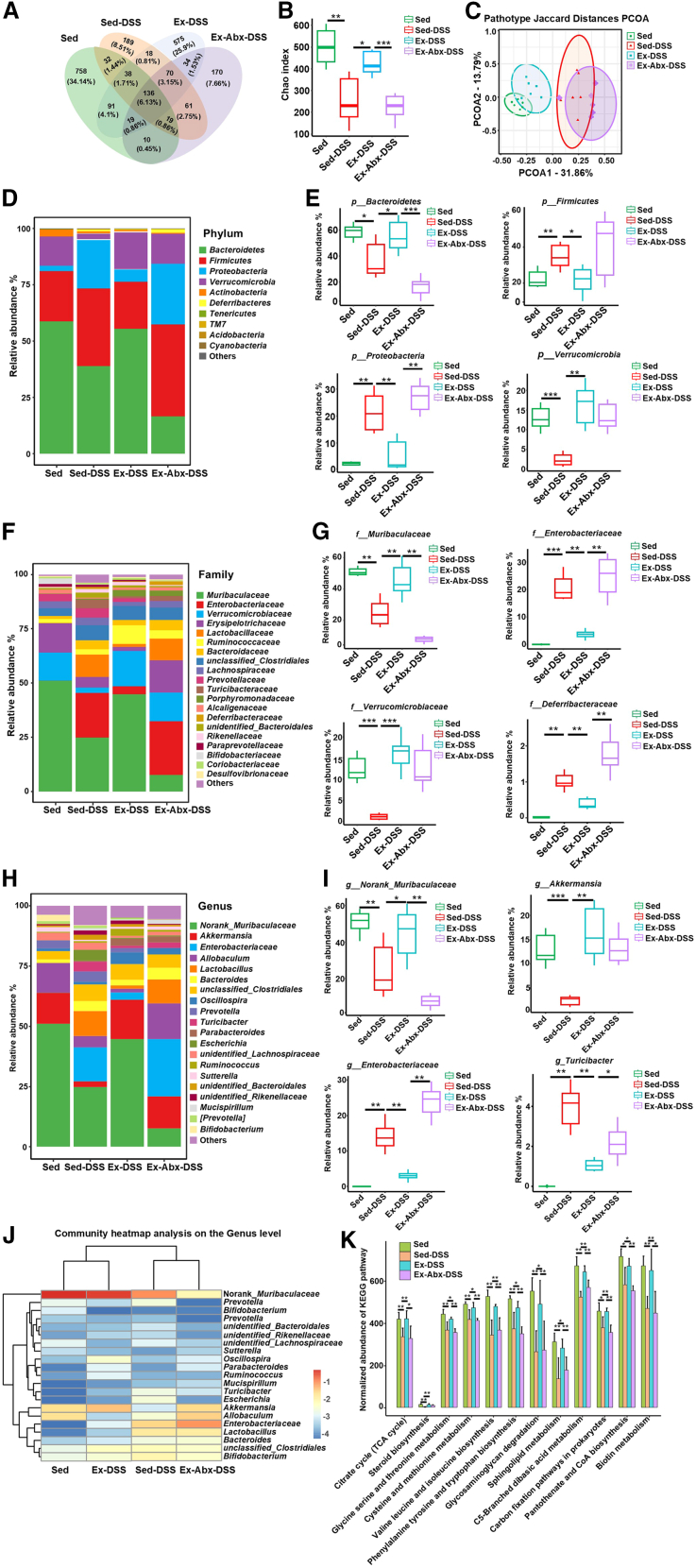
Voluntary exercise reshapes microbiota composition and enriches metabolic pathways (A) Venn diagram of the average numbers of operational taxonomic units (OTUs) and overlapping OTUs in different mouse groups. (B) The α diversity analysis via the Chao index. (C) The β diversity via principal coordinate analysis (PCoA). (D and E) Relative abundance of microbes at the phylum level. (F and G) Relative abundance of microbes at the family level. (H–J) Microbial relative abundance at the genus level. (K) Kyoto Encyclopedia of Genes and Genomes metabolic pathway analysis among the groups. Data are presented as means ± standard deviations, *n* = 6 per group, ∗*p* < 0.05, ∗∗*p* < 0.01, and ∗∗∗*p* < 0.001. DSS, dextran sulfate sodium; Sed, sedentary control; Sed-DSS, sedentary DSS-treated mice; Ex-DSS, exercised DSS-treated mice; Abx, antibiotics.

Since the intestinal barrier plays a crucial role in colitis pathogenesis, we next examined the effects of exercise on intestinal barrier integrity (Figure 3). Immunofluorescence analyses revealed that exercise prevented DSS-induced loss of tight junction proteins zonula occludens-1 (ZO-1) and occludin (Figure 3A). Both protein (Figures 3B and 2C) and mRNA levels (Figure 3D) of these markers were significantly higher in Ex-DSS mice (ZO-1: *p* = 0.001 and *p* < 0.001; occludin: *p* = 0.001 and *p* < 0.001) than in Sed-DSS mice. However, antibiotic treatment (Ex-Abx-DSS) markedly blunted these benefits (*p* < 0.001, *p* = 0.007, *p* = 0.003, and *p* = 0.004). Furthermore, fluorescein isothiocyanate (FITC)-FD4 assays demonstrated reduced intestinal permeability (Figure 4E), and Alcian blue-periodic acid Schiff staining confirmed better preservation of goblet cells (Figure 3F) in exercised mice, which was diminished following microbial depletion. Exercise enhanced intestinal barrier integrity through mechanisms that rely on a balanced gut microbiota.

### Exercise enhanced gut microbiota diversity and modulated metabolic pathways

Next, we evaluated the impact of exercise on gut microbiome composition via 16S ribosomal RNA (rRNA) sequencing of fecal samples (Figure 4). A Venn diagram (Figure 4A) shows the distribution of operational taxonomic units across the groups. In Figure 4B, the Sed-DSS group exhibited significantly reduced α-diversity (Chao index, *p* = 0.005) compared to the Sed group, whereas exercise (Ex-DSS) partially restored microbial richness (*p* = 0.025). This improvement was negated in the Ex-Abx-DSS group (*p* < 0.001). Principal coordinate analysis of β diversity (Figure 4C) demonstrated that Ex-DSS samples clustered closer to the Sed group, whereas Ex-Abx-DSS samples diverged markedly.

At the taxonomic level, exercise-induced modulation was evident across multiple tiers. At the phylum level (Figures 4D and 3E), Ex-DSS mice showed an increased relative abundance of beneficial phyla, such as Bacteroidetes and Verrucomicrobia, coupled with a decrease in Firmicutes and Proteobacteria, compared to Sed-DSS mice. At the family (Figures 4F and 4G) and genus levels (Figures 4H–4J), exercise significantly reduced the prevalence of potentially pathogenic taxa (e.g., Enterobacteriaceae and Turicibacter) while preserving beneficial groups (e.g., norank*_*Muribaculaceae and Akkermansia). These taxonomic shifts were reversed in the Ex-Abx-DSS group, further highlighting the dependence of these beneficial changes on intact microbial communities.

Kyoto Encyclopedia of Genes and Genomes (KEGG) pathway analysis provided further insight into the functional implications of these microbial changes (Figure 4K). In Ex-DSS mice, exercise significantly enriched the pathways involved in energy metabolism (such as the citrate cycle), amino acid synthesis (including glycine, serine, and threonine metabolism), and immune regulation. These functional enhancements were absent in the Ex-Abx-DSS group, indicating that the metabolic potential induced by exercise is contingent on the maintenance of microbial integrity.

### Voluntary exercise suppresses NET formation via gut microbiota modulation

**Figure 5:**
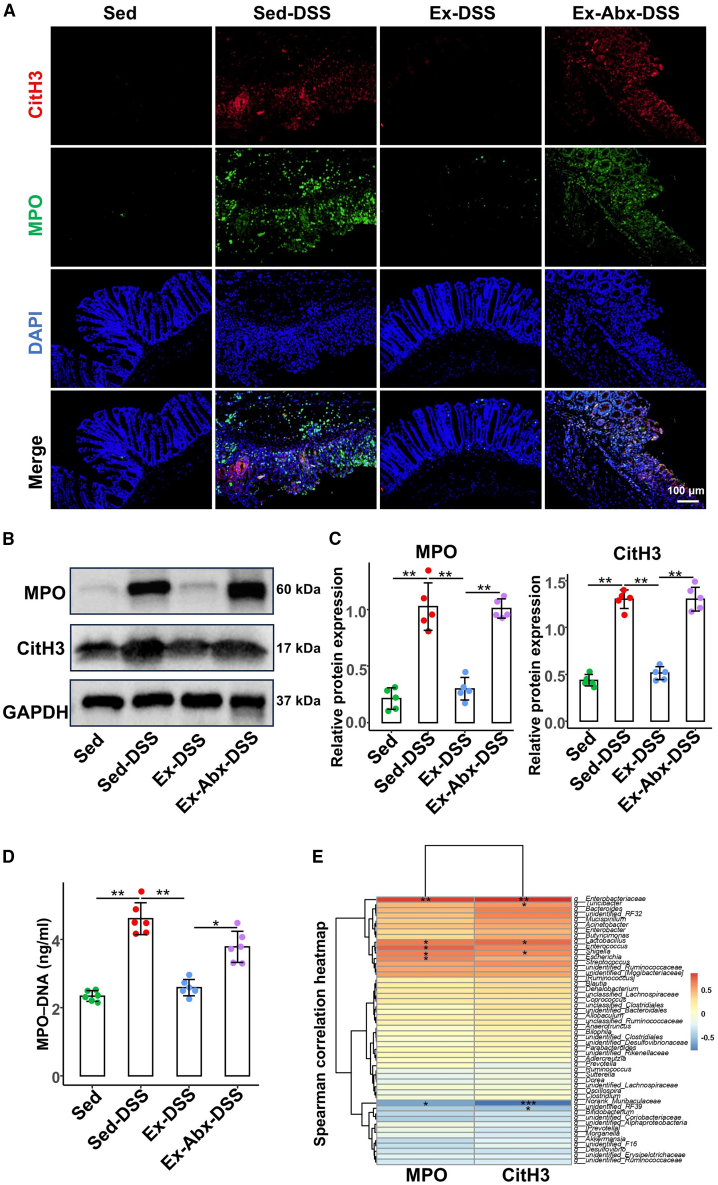
Voluntary exercise inhibits NET formation in a manner that is dependent on a balanced gut microbiota (A) Representative images show NET formation of merged CitH3 (red), MPO (green), and DNA (blue) of colon sections via immunofluorescence. (B) The protein levels of colonic CitH3 and MPO are evaluated by western blot. (C) Relative protein expressions of CitH3 and MPO in colon (*n* = 5 per group). (D) MPO-DNA complex levels in plasma are detected by enzyme-linked immunosorbent assay. (E) The correlation heatmap of genera and the expression of CitH3 and MPO protein. Data are presented as means ± standard deviations, ∗*p* < 0.05 and ∗∗*p* < 0.01. NET, neutrophil extracellular trap; MPO, myeloperoxidase; DSS, dextran sulfate sodium; Sed, sedentary control; Sed-DSS, sedentary DSS-treated mice; Ex-DSS, exercised DSS-treated mice; Abx, antibiotics.

Given the role of NETs in the pathogenesis of colitis, we assessed NET formation across the experimental groups. Immunofluorescence analysis (Figure 5A) revealed a significant increase in NET-related MPO and citrullinated histone H3 (CitH3) protein colocalization in the Sed-DSS group compared to that in the Sed group, whereas this colocalization was markedly reduced in the Ex-DSS group. However, in the Ex-Abx-DSS group, in which the gut microbiota was depleted, colocalization increased relative to that in the Ex-DSS group, suggesting that the gut microbiota is critical for exercise-mediated NET suppression. These findings were further corroborated by western blot analysis (Figures 5B and 5C). Consistently, enzyme-linked immunosorbent assay (ELISA) results (Figure 5D) showed elevated plasma MPO-DNA complexes in the Sed-DSS group (*p* = 0.001), which were significantly reduced by exercise in the Ex-DSS group (*p* = 0.002) but increased in the Ex-Abx-DSS group (*p* = 0.023). Exercise effectively suppressed NET formation in the inflamed colon, an effect that is dependent on gut microbiota, as antibiotic-induced microbiota depletion abolishes this suppression, emphasizing the essential role of the gut microbiota in exercise-mediated NET inhibition.

Spearman’s correlation analysis was performed to evaluate the relationship between alterations in the gut microbiota and NET-associated protein expression. As shown in Figure 5E, Enterobacteriaceae, Lactobacillus, and Shigella were positively correlated with MPO and CitH3 expression levels. An increase in beneficial bacteria (e.g., norank*_*Muribaculaceae) was inversely correlated with NET protein expression.

### Exercise-derived FMT recapitulated protection

**Figure 6:**
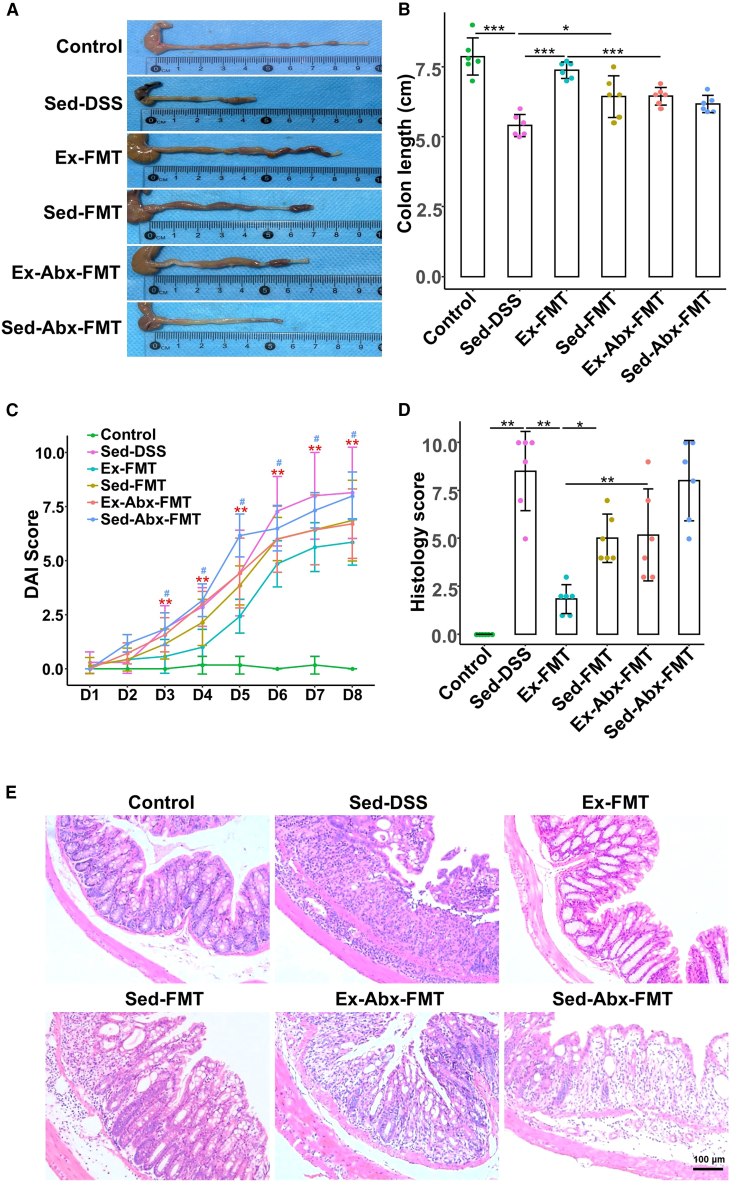
FMT from voluntary exercise-treated mice into receipt mice ameliorates colitis (A and B) Colon length of mice. (C) Clinical disease activity index scores of each group. (D) Histological scores. (E) Representative hematoxylin and eosin staining images of colonic sections at 200× magnification, scale bars: 100 μm. Data are presented as means ± standard deviations, *n* = 6 per group. Red ∗ and ∗∗ represent crosswise comparison between the Sed-DSS and control groups with *p* < 0.05 and *p* < 0.01, respectively. Blue ^#^ and ^##^ represent crosswise comparison of the Ex-FMT and Sed-DSS groups with *p* < 0.05 and *p* < 0.01, respectively. FMT, fecal microbiota transplantation; DSS, dextran sulfate sodium; Sed-DSS, sedentary DSS-treated mice; Sed-FMT, DSS-treated mice received FMT from sedentary mice; Ex-FMT, DSS-treated mice received FMT from exercised mice; Abx, antibiotics.

To further validate the role of gut microbiota in exercise-mediated benefits, we conducted FMT experiments in six groups of sedentary recipients as follows: control (control mice without fecal supernatants), Sed-DSS (DSS-treated mice without fecal supernatants), Ex-FMT (DSS-treated mice subjected to fecal supernatants from exercised mice), Sed-FMT (DSS-treated mice subjected fecal supernatants from sedentary mice), Ex-Abx-FMT (DSS-treated mice subjected fecal supernatants from exercised mice after antibiotic treatment), and Sed-Abx-FMT (DSS-treated mice subjected to fecal supernatants from sedentary mice after antibiotic treatment). For the experimental workflow, please see Experiment 2 in Figure 1 (located in the STAR Methods section). As shown in Figures 6A and 6B, the Sed-DSS group had shorter colons than the control group (*p* < 0.001), whereas the Ex-FMT and Sed-FMT groups had longer colons (*p* < 0.001 and *p* = 0.018, respectively). The Ex-FMT group showed greater recovery and near-control levels. By contrast, colon length was significantly reduced in both the Ex-Abx-FMT and Sed-Abx-FMT groups. Additionally, the Sed-DSS group had higher DAI (Figure 6C) (*p* = 0.001) and histological scores (Figures 6D and 6E) (*p* = 0.002), which were significantly lower in the Ex-FMT group (*p* = 0.023 and *p* = 0.004, respectively) and slightly lower in the Sed-FMT group (*p* = 0.26 and *p* = 0.018, respectively) but increased in the Ex-Abx-FMT and Sed-Abx-FMT groups, approaching Sed-DSS levels.

**Figure 7:**
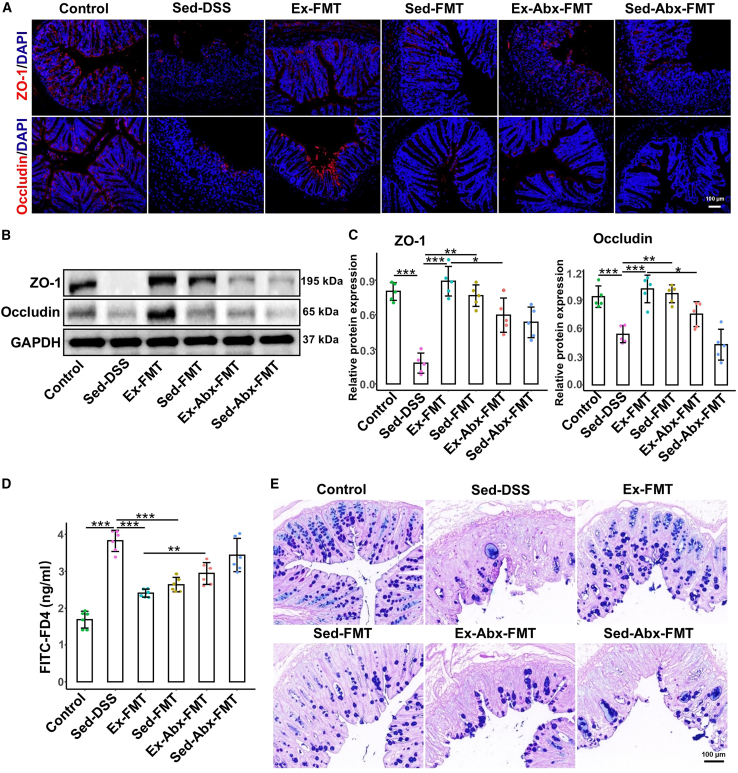
FMT from voluntary exercise-treated mice maintains intestinal barrier integrity of receipt mice (A) The distribution of ZO-1 and occludin in colonic tissues is observed by immunofluorescence staining, scale bars: 100 μm. (B) The protein levels of colonic ZO-1 and occludin were evaluated by western blotting. (C) Relative protein expressions of ZO-1 and occludin in the colon (*n* = 5 per group). (D) The intestinal permeability was analyzed via oral gavage of FITC-FD4. (E) The number of goblet cells in the colon is visualized by AB-PAS staining. Data are presented as means ± standard deviations, ∗*p* < 0.05, ∗∗*p* < 0.01, and ∗∗∗*p* < 0.001. FMT, fecal microbiota transplantation; DSS, dextran sulfate sodium; Sed, sedentary control; Sed-DSS, sedentary DSS-treated mice; Ex-DSS, exercised DSS-treated mice; Sed-FMT, DSS-treated mice received FMT from sedentary mice; Ex-FMT, DSS-treated mice received FMT from exercised mice; Abx, antibiotics.

Immunofluorescence in Figure 7A shows reduced ZO-1 and occludin distribution in the Sed-DSS group. Both Ex-FMT and Sed-FMT prevented this reduction, with Ex-FMT exerting a stronger effect. However, Ex-Abx-FMT and Sed-Abx-FMT did not prevent this decline. Western blot analysis (Figures 7B and 7C) revealed significantly higher ZO-1 and occludin levels in the Ex-FMT (*p* < 0.001 and *p* < 0.001, respectively) and Sed-FMT (*p* = 0.002 and *p* = 0.007, respectively) groups than in the Sed-DSS group, with a greater increase in the Ex-FMT group. By contrast, Ex-Abx-FMT and Sed-Abx-FMT mice exhibited reduced expression levels. Additionally, FD4 permeability significantly decreased in Ex-FMT (*p* < 0.001) and moderately decreased in Sed-FMT (*p* < 0.001), whereas Ex-Abx-FMT and Sed-Abx-FMT showed a marked increase (Figure 7D). The number of goblet cells was notably reduced in Sed-DSS mice but increased in Ex-FMT mice, with a smaller increase observed in Sed-FMT mice (Figure 7E). However, neither the Ex-Abx-FMT nor Sed-Abx-FMT groups showed significant restoration of goblet cells.

**Figure 8:**
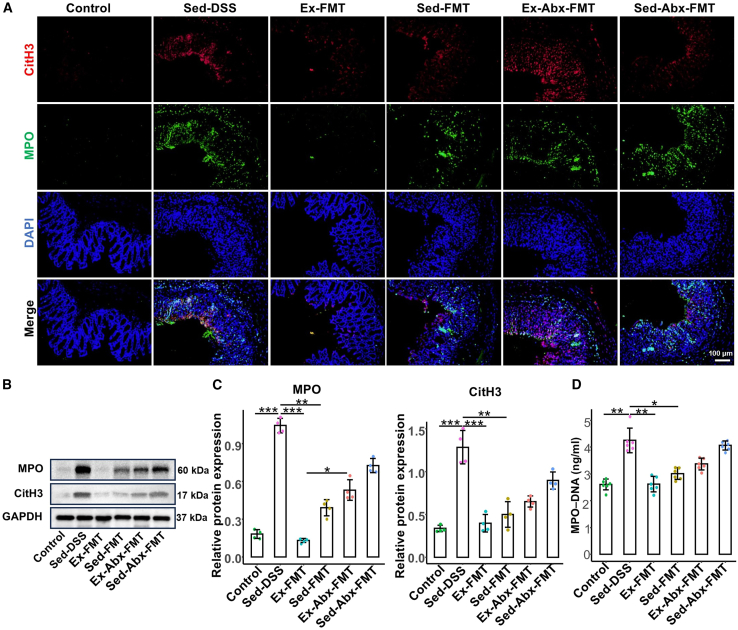
FMT from voluntary exercise-treated mice inhibits NET formation of receipt mice (A) Representative images show NET formation of merged CitH3 (red), MPO (green), and DNA (blue) of colon sections via immunofluorescence. (B) The protein levels of colonic CitH3 and MPO were evaluated by western blotting. (C) Relative protein expressions of CitH3 and MPO in the colon (*n* = 4 per group). (D) MPO-DNA complex levels in plasma are detected by enzyme-linked immunosorbent assay. Data are presented as means ± standard deviations, ∗*p* < 0.05, ∗∗*p* < 0.01, and ∗∗∗*p* < 0.001. FMT, fecal microbiota transplantation; DSS, dextran sulfate sodium; Sed, sedentary control; Sed-DSS, sedentary DSS-treated mice; Ex-DSS, exercised DSS-treated mice; Sed-FMT, DSS-treated mice received FMT from sedentary mice; Ex-FMT, DSS-treated mice received FMT from exercised mice; Abx, antibiotics; NET, neutrophil extracellular trap; MPO, myeloperoxidase.

Figure 8A shows the immunofluorescence colocalization of the NET-related proteins CitH3 and MPO in the colon. The Sed-DSS group showed significantly higher colocalization than the control group. Both Ex-FMT and Sed-FMT reduced this colocalization, with a stronger reduction observed in the Ex-FMT group. However, Ex-Abx-FMT or Sed-Abx-FMT did not reduce these levels. Western blot analysis of CitH3 and MPO (Figures 8B and 8C) showed similar trends. ELISA results (Figure 8D) revealed a significant increase in MPO-DNA levels in the Sed-DSS group, which was markedly reduced by Ex-FMT and, to a lesser extent, by Sed-FMT, whereas the Ex-Abx-FMT and Sed-Abx-FMT groups showed no reduction.

### PAD4 inhibition synergized with exercise to reduce colitis severity and NET formation

**Figure 9:**
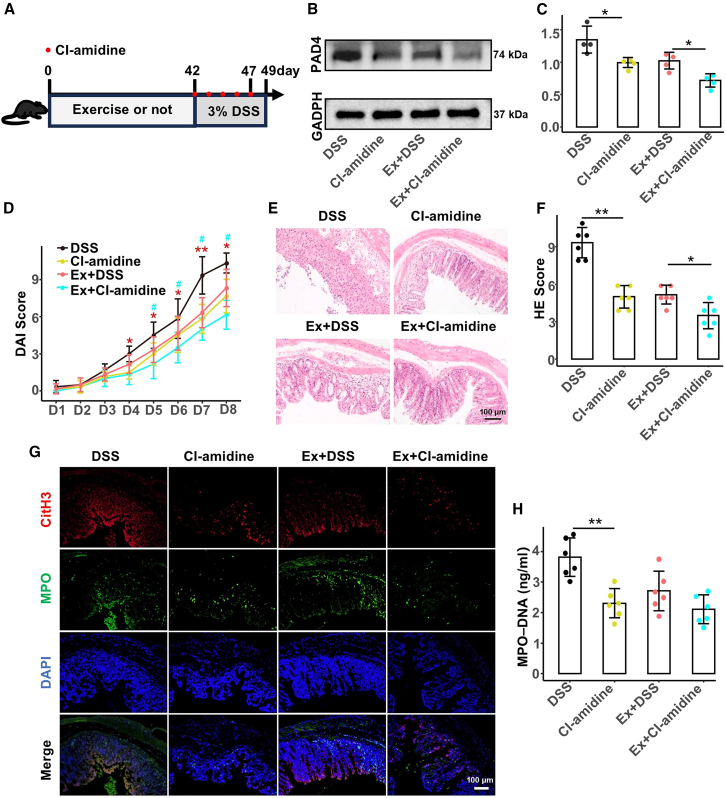
Cl-amidine enhances the protective effects of exercise by further inhibiting PAD4-mediated NET formation, thereby attenuating colonic inflammation (A) Mice subjected to sedentary or 6-week exercise regimens received daily intraperitoneal Cl-amidine injections during days 1–5 of 3% DSS-induced colitis. (B and C) Western blot analysis of PAD4 in colon tissue (*n* = 4 per group). (D) Colitis severity was evaluated using disease activity index scores from days 1–8, *n* = 6 per group. (E and F) Representative hematoxylin and eosin staining of colonic tissue and corresponding histopathological scores on day 8 following DSS treatment. (G) Representative immunofluorescence images illustrate NET formation in colonic sections, with merged signals for CitH3 (red), MPO (green), and DNA (blue). (H) NET release was quantified in plasma by measuring MPO-DNA complex levels using enzyme-linked immunosorbent assay. Data are presented as means ± standard deviations, red ∗∗ and ∗ represent crosswise comparison between the DSS and Cl-amidine groups at *p* < 0.01 and *p* < 0.05, respectively; blue ## and # represent crosswise comparison of the Cl-amidine and Ex + Cl-amidine groups with *p* < 0.01 and *p* < 0.05, respectively. MPO, myeloperoxidase; DSS, dextran sulfate sodium; Ex + DSS, exercised DSS-treated mice; NET, neutrophil extracellular trap.

We administered the PAD4 inhibitor Cl-amidine to further elucidate the role of NETs in exercise-induced protection against colitis (Figure 9A). Western blot analysis confirmed that Cl-amidine significantly reduced PAD4 expression in the colons of DSS-treated mice (Figures 9B and 9C). Importantly, mice receiving both exercise and Cl-amidine exhibited a further reduction in PAD4 levels compared with those treated with either intervention alone, suggesting a synergistic effect. The severity of colitis was evaluated using DAI scores and histopathological analysis. Cl-amidine treatment lowered the DAI scores from day 4 onward, with combination therapy (exercise + Cl-amidine) showing pronounced improvement from day 5 onward (Figures 9D–9F). Histological examination revealed that the combination treatment effectively alleviated epithelial disruption, glandular necrosis, and inflammatory cell infiltration. Moreover, immunofluorescence and ELISA assays showed that Cl-amidine significantly decreased the number of CitH3- and MPO-expressing cells in the colon, as well as circulating MPO-DNA complexes (Figures 9G and 9H). Thus, PAD4 inhibition and exercise synergistically suppressed NET formation and ameliorated colonic inflammation.

## Discussion

Our study demonstrated that 6-week voluntary exercise preconditioning markedly attenuated DSS-induced colitis. Exercise improved colitis by modulating intestinal microbiota, inhibiting NET formation, and preserving intestinal barrier integrity. The abolition of these benefits following antibiotic-induced microbiota depletion underscores the indispensable role of intact microbial communities in exercise-induced protection. Furthermore, the replication of these benefits via FMT in exercised donors confirmed that exercise-induced alterations in the gut microbiota directly contributed to improved mucosal barrier function and immune regulation. Moreover, pharmacological inhibition of PAD4 further enhanced these protective effects, underscoring the importance of the PAD4-NET axis in the pathogenesis of colitis.

Despite significant advancements in anti-inflammatory therapies for UC, current treatments yield only moderate improvements, underscoring the urgent need for adjunct strategies that not only attenuate colonic inflammation but also mitigate the side effects of conventional treatments.^3^^,^^5^ Our findings highlight exercise and exercise-derived FMT as viable adjunct therapies that target both the microbial and immune pathways, addressing this unmet clinical need. Exercise has demonstrated broad anti-inflammatory benefits in various inflammatory diseases. Recent studies have indicated that voluntary exercise can reduce inflammation and symptoms in DSS-induced colitis models.^35^^,^^36^ Using a similar voluntary exercise regimen, our results showed that 6 weeks of voluntary exercise significantly prevented and reduced the severity of DSS-induced colitis, as evidenced by reduced colon shortening, lower DAI scores, and improved histological outcomes. These results corroborate previous findings and confirm that voluntary exercise effectively protects against DSS-induced colonic inflammation.^16^ Importantly, these benefits were abolished by broad-spectrum antibiotic treatment, which resulted in significantly shorter colons, higher DAI scores, and more pronounced histological damage, highlighting the essential role of balanced gut microbiota in mediating anti-inflammatory effects. Moreover, disruption of the intestinal mucosal barrier, which is a critical component of colitis pathogenesis, predisposes the gut to inflammation and microbial translocation.^2^ Consistent with previous studies,^37^ our current study confirmed that voluntary exercise enhances intestinal barrier stability by preserving tight junction proteins such as ZO-1 and occludin and decreasing intestinal permeability. The barrier-protective effects of exercise were nearly eliminated following antibiotic-induced microbial depletion, reinforcing the interdependence of microbial homeostasis and barrier integrity in mitigating colonic inflammation.

Exercise-induced modulation of the gut microbiota plays a critical role in mediating therapeutic effects. Dysbiosis—characterized by reduced microbial diversity and an imbalanced Firmicutes-to-Bacteroidetes ratio (F/B ratio)—is a well-established pathogenic mechanism in IBD and DSS-induced colitis models.^38^^,^^39^^,^^40^ Our 16S rRNA sequencing analyses revealed that sedentary mice with DSS-induced colitis exhibited significant dysbiosis, including significantly reduced microbial diversity, elevated F/B ratio, and increased abundance of pro-inflammatory taxa such as Enterobacteriaceae and Turicibacter (Figures 4B–4I). However, 6 weeks of voluntary exercise partially restored microbial diversity and maintained a balanced F/B ratio. Exercise curtailed the enrichment of harmful bacteria, such as Enterobacteriaceae and Turicibacter, while preserving beneficial taxa, including norank*_*Muribaculaceae and Akkermansia, which produce short-chain fatty acids (SCFAs), such as butyrate, that inhibit NET formation and reinforce barrier integrity.^41^^,^^42^^,^^43^ Our findings are consistent with those of Varghese et al.,^44^ who reported that moderate exercise promotes microbial diversity and SCFA production, thereby supporting mucosal barrier function and immune balance. We further utilized KEGG metabolic pathway analysis to predict potential microbial community functions, revealing that exercise-induced microbial shifts were associated with enhanced metabolic pathways, particularly amino acid biosynthesis and sphingolipid metabolism (Figure 4K), which regulate immune function and epithelial homeostasis.^45^^,^^46^^,^^47^ Notably, antibiotic-induced depletion of the gut microbiota disrupted this microbial balance, markedly reducing Bacteroidetes and norank*-*Muribaculaceae while enriching Proteobacteria and Enterobacteriaceae. This dysbiosis abolished the exercise-mediated suppression of NET formation, as evidenced by re-elevated levels of protein CitH3, MPO, and MPO-DNA complexes in exercised colitis mice following antibiotic treatment. Dysbiosis-induced immune dysregulation has been well documented as a key driver of aberrant NET formation in various inflammatory conditions.^26^^,^^48^ The depletion of those beneficial taxa and the expansion of harmful bacteria after antibiotic treatment may have an adverse effect on immune regulation, thereby exacerbating the formation of NETs. These findings reaffirm the indispensable role of balanced gut microbiota in mediating exercise-induced benefits.

The gut microbiota plays a crucial role in regulating the intestinal immune response.^40^ Recent reports have documented elevated NET levels in both patients with IBD and in experimental colitis models, where excessive NETs (marked by increased MPO and CitH3 levels) impair barrier permeability and exacerbate tissue damage.^25^^,^^49^ Consistent with these findings,^50^^,^^51^ our data showed that voluntary exercise significantly reduced NET-associated protein expression and circulating MPO-DNA complexes in DSS-induced colitis. Importantly, this effect was largely abolished by antibiotic-induced microbiota depletion, highlighting the microbiota-dependent mechanism that regulates NET formation. Furthermore, our correlation analysis revealed an inverse relationship between the abundance of beneficial bacteria (such as norank*_*Muribaculaceae) and NET expression, suggesting that an increased abundance of certain beneficial bacteria resulting from exercise may attenuate excessive neutrophil activation and downregulate NET formation. Collectively, these findings imply that exercise mitigates colonic inflammation through a dual mechanism, directly suppressing NET formation and indirectly promoting healthier, metabolically active gut microbiota.

Considering the crucial role of the gut microbiota in mediating the beneficial effects of exercise, we next evaluated whether transferring the microbiota from exercised donors could replicate these protective outcomes. Mice receiving microbiota from exercised donors exhibited reduced colonic inflammation and enhanced barrier integrity compared with those receiving microbiota from sedentary donors. These results are consistent with those of previous reports showing that FMT from healthy donors can ameliorate colitis.^20^ However, our work uniquely highlights that this protective effect was nullified when the microbiota from exercised donors was depleted with antibiotics prior to transplantation, underscoring the necessity of a healthy microbial community for the observed benefits. The mechanisms underlying these effects may involve the suppression of NET formation, mediated by enriched beneficial bacterial populations, such as norank*_*Muribaculaceae and Akkermansia, preserved in exercised donors, which likely contribute to enhanced mucosal integrity.^52^^,^^53^^,^^54^ Although previous studies have reported variable efficacy of FMT in colitis models, our data emphasize that donor characteristics, particularly exercise-induced microbial profiles, are crucial determinants of therapeutic success. This insight not only aligns with the existing literature but also deepens our understanding by indicating that targeted modulation of donor microbiota via exercise can enhance FMT outcomes.

Building on our finding that exercise-induced microbial alterations contribute to reduced NET formation and improved colonic health, we explored the role of PAD4 inhibition, which is a critical driver of NET release. PAD4 facilitates NET formation by citrullinating histones, which leads to chromatin decondensation. In our DSS-induced colitis model, the administration of Cl-amidine, a PAD4 inhibitor, significantly reduced disease activity and histopathological damage in sedentary mice, yielding improvements comparable to those observed with exercise. Combining Cl-amidine treatment with exercise resulted in the most pronounced attenuation of both colitis severity and NET formation, suggesting a synergistic enhancement of the anti-inflammatory effects. These observations underscore the pivotal role of the PAD4-NET axis in the pathogenesis of colitis and highlight the potential of integrated therapeutic strategies that combine physical activity with targeted PAD4 inhibition.

In conclusion, our research provides compelling evidence that voluntary exercise confers robust protection against DSS-induced colitis through interconnected mechanisms, including restoration of gut microbiota diversity, enhancement of intestinal barrier integrity, and suppression of NET formation. Our findings are unique in demonstrating that these beneficial effects are microbiota dependent, as evidenced by their loss upon antibiotic treatment and that they can be partially transferred via FMT from exercised donors. Furthermore, the synergistic benefits of PAD4 inhibition underscore the critical role of the PAD4-NET axis and highlight the potential of combining physical activity with targeted molecular interventions to achieve superior anti-inflammatory outcomes. Future clinical trials should explore whether exercise-derived FMT can benefit patients with limited mobility by providing microbiota-based therapeutic alternatives. Collectively, these findings provide novel mechanistic insights into the interplay among exercise, gut microbiota, and immune responses, suggesting that exercise-derived FMT may serve as a promising adjunct therapeutic strategy for UC, particularly in patients unable to engage in regular physical activity. Future studies should focus on identifying the specific microbial strains and metabolites responsible for these protective effects and validating these integrated strategies in clinical settings.

### Limitations of the study

Although our findings highlight the therapeutic potential of exercise-derived FMT, several limitations remain. First, we employed only a single exercise protocol, as previously described, without gradient comparisons of duration or intensity. The role of gut microbiota revealed by FMT from sedentary mice to exercised mice is worth studying. Clinical trials are needed to confirm these preclinical results. Second, the translation of exercise-derived FMT into clinical practice requires careful consideration of ethical concerns, patient safety, and stability of transplant outcomes. Third, although our study demonstrated that exercise-induced alterations in gut microbial communities and their metabolic pathways contribute to colitis control and NET inhibition, further research is needed to isolate the specific microbial strains and metabolites (e.g., SCFAs) responsible for these effects. Lastly, regarding exerkines—signaling molecules secreted in response to exercise—the complexity and variability in their expression (due to differences in exercise intensity, duration, and frequency) necessitate future studies to identify key myokines and to elucidate the gut-muscle axis in mediating exercise benefits.^55^^,^^56^

## Resource availability

### Lead contact

For further information and requests for resources and reagents, please reach out to the lead contact, Bao Ping Yu (yubp62@163.com).

### Materials availability

This study did not generate new unique reagents.

### Data and code availability

• The 16 S rRNA raw sequencing data have been deposited at NCBI Sequence Read Archive at https://www.ncbi.nlm.nih.gov/bioproject/1184370 and are publicly available. Accession numbers are listed in the key resources table. Original western blot images have been deposited at Mendeley (https://data.mendeley.com/datasets/8vpx33d8dw/1) and are publicly available. Microscopy data reported in this paper will be shared by the lead contact upon request.
• This paper does not report original code.
• Any additional information required to reanalyze the data reported in this paper is available from the lead contact upon request.

## Acknowledgments

This study was supported by the 10.13039/501100001809National Natural Science Foundation of China (grant no. 81770638) and Talent Start-Up Fund Project of 10.13039/501100014361Hubei University of Medicine (grant no. 2021QDJZR002).

## Author contributions

Conceptualization, B.Z. and B.Y.; methodology, B.Z. and H.W.; investigation, H.W., H.Z., Q.S., and Y.X.; writing – original draft, B.Z.; writing – review and editing, B.Z., H.W., and B.Y.; funding acquisition, H.W. and B.Y.; resources, H.Z., Q.S., and Y.X.; supervision, B.Y.

## Declaration of interests

The authors declare no competing interests.

## STAR★Methods

### Key resources table

**Table:** 

REAGENT or RESOURCE	SOURCE	IDENTIFIER
**Antibodies**		

Rabbit monoclonal anti-ZO1 tight junction	Abcam	Cat# ab276131
Rabbit monoclonal anti-Occludin	Abcam	Cat# ab216327
Rabbit polyclonal anti- Histone H3 (citrulline R2 + R8 + R17) (CitH3)	Abcam	Cat# ab5103
Rabbit polyclonal anti- peptidyl arginine deiminase 4 (PAD4)	Abcam	Cat# ab96758
Goat polyclonal anti- Myeloperoxidase (MPO)	R&D system	Cat# AF3667
Rabbit polyclonal anti- GAPDH	Proteintech	Cat# 10494-1-AP
HRP-conjugated Goat Anti-Rabbit IgG(H+L)	Proteintech	Cat# SA00001-2
HRP-conjugated Donkey Anti-Goat IgG(H+L)	Proteintech	Cat# SA00001-3
Alexa Flour-594 Donkey anti Rabbit IgG (HL)	AntGene	Cat# ANT030
Alexa Flour-488 Donkey anti Goat IgG (HL)	AntGene	Cat# ANT025

**Biological samples**		

Mouse fecal samples	This paper	NCBI Sequence Read Archive (SRA): PRJNA1184370) https://www.ncbi.nlm.nih.gov/bioproject/1184370

**Chemicals, peptides, and recombinant proteins**		

Dextran sulfate sodium salt, colitis grade (36,000–50,000) (DSS)	MP Biomedical	SKU:02160110-CF
Vancomycin	MedChem Express	Cat# HY-B0671
Ampicillin	MedChem Express	Cat# HY-B0522
Neomycin	MedChem Express	Cat# HY-150520
Metronidazole	MedChem Express	Cat# HY-B0318
FD4 (FITC-dextran, MW 4000)	Sigma-Aldrich	Cat# 60842-46-8
RIPA lysis buffer	Beyotime	Cat# P0013B
Phenylmethanesulfonyl fluoride (PMSF)	Beyotime	Cat# ST506
Protease inhibitor cocktail	Beyotime	Cat# P1006
TRIzol reagent	Invitrogen	Cat# A33250
DAPI dihydrochloride	Beyotime	Cat# C1005
Cl-amidine	GLPBIO	Cat# GC35706

**Critical commercial assays**		

Cell death detection ELISA kit	Roche	Cat# 11774425001
Enhanced BCA Protein Assay Kit	Beyotime	Cat# P0010S
PrimeScript™ Reverse Transcription reagent Kit	TaKaRa	Cat# RR037A
SYBR-Green PCR Master Mix Kit	TaKaRa	Cat# RR820A
Enhanced chemiluminescence (ECL) reagent	Abbkine	Cat# BMU101-EN

**Deposited data**		

Source data	This paper	https://data.mendeley.com/datasets/8vpx33d8dw/1

**Experimental models: Organisms/strains**		

Mouse: C57Bl/6J	Vital River Laboratory (Beijing, China)	N/A

**Oligonucleotides**		

Primers, See Table S1	This paper	N/A

**Software and algorithms**		

GraphPad Prism 8.0	GraphPad Software	https://www.graphpad.com/
R version 4.2.3	R Development Core Team	https://www.r-project.org
ImageJ	ImageJ	https://imagej.nih.gov/ij/
PICRUSt2	PICRUSt Software	https://github.com/picrust/picrust2

### Experimental model and study participant details

#### Experimental animals

Six-week-old male C57Bl/6J mice were obtained from the Vital River Laboratory (Beijing, China) and acclimatized for 1 week in a specific pathogen-free environment. The mice were housed under controlled conditions (22°C, 12-h light/dark cycle) at the Animal Experiment Center, Renmin Hospital of Wuhan University (Wuhan, China) and subsequently randomly assigned to experimental groups. All procedures were approved by the Laboratory Animal Welfare and Ethics Committee of the Renmin Hospital of Wuhan University (P.R. China, IACCU issue no.: 202200156).

#### Experimental design

The grouping and experimental procedures are shown as follows (Figure 1):

(1) **Voluntary exercise model and DSS-induced acute colitis model were established to explore whether voluntary exercise could protect the gut barrier in acute colitis.** The mice were randomly divided into the exercise and sedentary groups (n = 16 per group). Mice in the exercise group were housed individually with unrestricted access to a telemetric running wheel (20 cm diameter), whereas sedentary mice were housed nearby without running wheel access. Each running wheel was equipped with a device that recorded daily activity, revealing that the exercise group ran an average of 6.61 ± 0.06 km per day. Detailed records of the exercise model are presented in Table 1. Sedentary mice were further divided into the sedentary (Sed) and DSS-induced colitis (Sed-DSS) subgroups. Similarly, exercised mice were divided into the DSS-induced colitis with exercise (Ex-DSS) and DSS-induced colitis with antibiotic-treated exercise (Ex-Abx-DSS) subgroups (n = 6–8 per group). During the final 7 days of the exercise phase, the microbiota in the Ex-Abx-D group was depleted using a broad-spectrum antibiotic cocktail (200 μL per mouse) administered every 12 h via oral gavage. Antibiotics (Abx) included vancomycin (100 mg/kg), ampicillin (200 mg/kg), neomycin (200 mg/kg), and metronidazole (200 mg/kg), following the protocol described by Chen et al.^57^ The other groups were provided normal water. Afterwards, all groups except the Sed group were administered 3% (w/v) DSS in their drinking water for 7 days, with the water replaced every other day. The Sed group received autoclaved drinking water.
(2) **FMT was performed to investigate the role of gut microbiota in the effects of voluntary exercise.** Six-week-old mice were treated with above-mentioned Abx via oral gavage (200 μL per mouse) every 12 h for 7 days to create germ-free recipients. The mice were randomly assigned to six groups (n = 6 per group) as follows: Control, Sed-DSS, Ex-FMT (fecal supernatants from exercised mice), Sed-FMT (fecal supernatants from sedentary mice), Ex-Abx-FMT (fecal supernatants from exercised mice after Abx treatment), and Sed-Abx-FMT (fecal supernatants from sedentary mice after Abx treatment). For transplantation, fecal supernatant was administered by oral gavage (0.2 mL per mouse) daily for 2 consecutive weeks, as previously described. The Control and Sed-DSS groups received the vehicle (phosphate-buffered saline [PBS] with 20% glycerol) following the same method. Acute colitis was induced using 3% DSS for 7 days. Throughout this period, the mice were weighed daily, and stool consistency and rectal bleeding were monitored to calculate DAI scores as described previously. At the end of DSS treatment, all mice were sacrificed, and colon length was measured. Colon tissues were rinsed in PBS, with half fixed in 4% paraformaldehyde and the rest rapidly frozen in liquid nitrogen, then stored at −80°C.
(3) **Cl-amidine was administered to assess the role of PAD4-mediated NET formation in colitis pathogenesis and exercise-induced protection.** This regimen was designed to suppress NET formation during the acute phase of colitis. Mice were divided into the Sed-DSS, Ex-DSS, Sed-DSS + Cl-amidine, and Ex-DSS + Cl-amidine subgroups. Mice that underwent 6 weeks of voluntary exercise, along with their sedentary counterparts, received daily intraperitoneal injections of the PAD4 inhibitor Cl-amidine (25 mg/kg/day) or vehicle (PBS) from days 1 to 5 during DSS administration.

### Method details

#### Fecal suspension preparation for transplantation

Fresh feces were collected from 6-week exercised and sedentary donor mice, with a second collection occurring 1 week after the administration of the afore mentioned antibiotics. Aqueous fecal extracts were prepared based on the method described by Chen et al., with appropriate modifications. Feces from each mouse within the same group were pooled and homogenized in sterile PBS with 20% glycerin in a 50 mL sterile tube (approximately 200 mg feces per 1 mL liquid). The mixture was then centrifuged at 800g for 5 min, and the resulting supernatant was aliquoted and stored at −80°C for later transplantation.

#### Intestinal permeability assessment

The *in vivo* intestinal permeability was assessed using FD4 (FITC-dextran, MW 4000). Mice in each group were fasted overnight with free access to water and then orally administered FD4 diluted in PBS (0.1 mg/g body weight). Plasma fluorescence intensity was quantified using a fluorescence spectrophotometre (485 nm/528 nm) 4 h after gavage. Plasma FD4 levels were determined using a standard curve of serial dilutions of FD4 in PBS.

#### Haematoxylin and eosin (H&E) and AB–PAS staining

The distal colon tissues were fixed in 4% paraformaldehyde for 24 h and embedded in paraffin. The paraffin-embedded colonic specimens were sectioned into approximately 4-μm slices, dewaxed in xylene, and rehydrated through a graded ethanol series before staining with H&E. Images were captured under a microscope and analyzed by a pathologist who was blinded to the experimental procedures, following previously described scoring criteria. The colonic segments were fixed in methanol–Carnoy solution (methanol: chloroform: glacial acetic acid = 6:3:1), paraffin embedded and sectioned into 3-μm serial slices. AB**–**PAS staining was performed to identify goblet cells according to the manufacturer’s instructions.

#### 16S rRNA sequencing and data analyses

Feces were collected from the mice in experiment I before sacrifice for microbiota analysis. Fecal microbial genomic DNA was extracted, and its quality was assessed according to the manufacturer’s instructions. The V3–V4 hypervariable region of the bacterial 16S rRNA gene was amplified by polymerase chain reaction (PCR) using universal primers. After quantifying PCR products and constructing libraries, sequencing was performed using an Illumina MiSeq platform. Microbial composition and diversity were analyzed, with α-diversity assessed using the Chao1 index and β-diversity visualized through PCoA based on the unweighted Unifrac distance. The relative abundances of specific phyla, families, and genera were identified, and functional microbial profiles were predicted using the PICRUSt software and annotated with the KEGG database.

#### MPO–DNA ELISA

To quantify NETs in plasma, MPO–DNA ELISA was performed. A 96-well ELISA plate was first coated with 5 μg/mL of anti-MPO antibody and incubated overnight at 4°C. After blocking with 1% bovine serum albumin (BSA), 20 μL of plasma samples and peroxidase-labelled anti-DNA monoclonal antibody were added to each well. The plates were incubated on a shaker at 300 rpm for 2 h, followed by three washes. After a 20-min incubation with the peroxidase substrate, the absorbance was measured at 405 nm.

#### Western blotting

Total protein was extracted from colon tissues using RIPA lysis buffer supplemented with phenylmethylsulfonyl fluoride and a protease inhibitor cocktail. Protein concentrations were measured using a bicinchoninic acid assay kit according to the manufacturer’s protocol. Equal amounts of proteins were separated by sodium dodecyl sulfate-polyacrylamide gel electrophoresis and transferred to polyvinylidene fluoride membranes. The membranes were blocked with 5% non-fat milk for 1 h at room temperature and incubated overnight at 4°C with primary antibodies: ZO-1, Occludin, CitH3, MPO, and glyceraldehyde-3-phosphate dehydrogenase (GAPDH). After washing, the membranes were incubated with secondary antibodies for 1 h at room temperature, and protein signals were detected using enhanced chemiluminescence on a Bio-Rad detection system.

#### Quantitative real-time PCR

Total RNA was extracted from colonic tissues using the TRIzol reagent. cDNA synthesis was performed using a Reverse Transcription kit, according to the manufacturer’s instructions. Real-time PCR was performed on a Bio-Rad CFX Connect™ Real-Time PCR Detection System with SYBR-Green PCR Master Mix. GAPDH served as an internal reference gene. The relative mRNA expression levels of target genes were quantified using the 2^−ΔΔCt^ method. The primer sequences are listed in Table S1.

#### Immunofluorescence

The paraffin-embedded sections were subjected to immunofluorescence staining after dewaxing and hydration. Antigens were retrieved under high pressure. After blocking with 3% BSA for 30 min, sections were incubated with primary antibodies overnight at 4°C. On the following day, the sections were washed thoroughly and incubated with Alexa Fluor 594-conjugated secondary antibody and/or Alexa Fluor 488-conjugated secondary antibodies for 1 h at room temperature. Nuclei were stained with 4,6-diamidino-2-phenylindole for 10 min. Finally, the slides were sealed with an anti-fluorescence quencher, and images were captured from five random fields at 200× magnification subsequently with a fluorescence microscope.

#### Statistical analysis

Statistical analyses were performed using statistical software R (R Foundation for Statistical Computing, version 3.5.3). We conducted the Shapiro-Wilk test for normality. P-values of most variables are less than 0.05 indicating not normally distributed. Paired samples Wilcoxon test was used to compare baseline and follow-up in the same animal. For the differences of between sub-groups, we performed Kruskal-Wallis Test to compare the differences between the multiple groups and found a significant difference. Then Mann-Whitney U-test was used for each two groups. Data are expressed as mean ± SEM. Statistical significance was defined as ∗p < 0.05, ∗∗p < 0.01, and ∗∗∗p < 0.001.
