# Supplementary material for: Gut microbiota from voluntary exercised mice protects the intestinal barrier by inhibiting neutrophil extracellular trap formation
Source: iScience. 2025 May 27;28(6):112763. doi: 10.1016/j.isci.2025.112763 (PMC12206135; doi:10.1016/j.isci.2025.112763)
Supplement: Document S1. Table S1 [file mmc1.pdf]

iScience, Volume *28*

Supplemental information

Gut microbiota from voluntary exercised mice protects the intestinal barrier by inhibiting neutrophil extracellular trap formation

Beibei Zhu, Hao Wu, Han Zhang, Qi Song, Yong Xiao, and Baoping Yu

Table S1. Primer sequences used for RT-PCR in this study.

Gene	Forward (5′–3′)	Reverse (5′–3′)
GAPDH	AGGTGGTGAAGCAGGCATCTGA	CGGCATCGAAGGTGGAAGAGTG
ZO-1	GAGATGTTTATGCGGACGGTG	TCCTCCATTGCTGTGCTCTTA
Occludin	GCCTTCTGCTTCATCGCTTCCT	ACACCATGATGCCCAGGATAGC
